# Parallel word processing in the flanker paradigm has a rightward bias

**DOI:** 10.3758/s13414-018-1547-2

**Published:** 2018-06-01

**Authors:** Joshua Snell, Jonathan Grainger

**Affiliations:** Laboratoire de Psychologie Cognitive & CNRS, Aix-Marseille Université, Marseille, France

**Keywords:** Reading, Attention, Orthography

## Abstract

Reading research is exhibiting growing interest in employing variants of the flanker paradigm to address several questions about reading. The paradigm is particularly suited for investigating parallel word processing, parafoveal-on-foveal influences, and visuospatial attention in a simple but constrained setting. However, this methodological deviation from natural reading warrants careful assessment of the extent to which cognitive processes underlying reading operate similarly in these respective settings. The present study investigated whether readers’ distribution of attention in the flanker paradigm resembles that observed during sentence reading; that is, with a rightward bias. Participants made lexical decisions about foveal target words while we manipulated parafoveal flanking words. In line with prior research, we established a parafoveal-on-foveal repetition effect, and this effect was increased for rightward flankers compared with leftward flankers. In a second experiment, we found that, compared with a no-flanker condition, rightward repetition flankers facilitated target processing, while leftward flankers interfered. Additionally, the repetition effect was larger for rightward than for leftward flankers. From these findings, we infer that attention in the flanker paradigm is indeed biased toward the right, and that the flanker paradigm thus provides an effective analogy to natural reading for investigating the role of visuospatial attention. The enhanced parafoveal-on-foveal effects within the attended region further underline the key role of attention in the spatial integration of orthographic information. Lastly, we conclude that future research employing the flanker paradigm should take the asymmetrical aspect of the attentional deployment into account.

For decades, cognitive psychology has employed the flanker paradigm (pioneered as the Eriksen flanker task by Eriksen & Eriksen, 1974) to investigate a multitude of things, such as attentional control, parallel stimulus processing, and response conflict (e.g., Eriksen & Eriksen, 1974; Gratton, Coles, & Donchin, 1992; Lamers & Roelofs, 2011; Schaffer & Laberge, 1979). The principal finding is that responses to target stimuli are influenced by task-irrelevant surrounding stimuli (e.g., a *left* response to a target arrow pointing left is slowed when this target is flanked by arrows pointing right), hence revealing human’s inability to effectively focus processing resources on the stimulus of interest without attending to irrelevant stimuli. Classically, those so-called noise stimuli were thought to impact on the stage of decision-making, with response conflict stemming from different stimuli relating to opposing responses (Eriksen & Eriksen, 1974). However, in a variant of the flanker paradigm wherein participants had to respond to the semantic category of target words, Schaffer and Laberge (1979) found that the semantic congruency rather than the response congruency of flankers influenced task performance (i.e., flanking words belonging to a different semantic category, but to the same response nonetheless increased response times). This result indicates that information may be integrated across words at the lexical or semantic level.

Now, more than 3 decades later, reading research is exhibiting renewed interest in the flanker paradigm, driven by the ongoing debate on whether words are processed serially (one-by-one) or in parallel during reading (Dare & Shillcock, 2013; Engbert & Kliegl, 2011; Engbert, Nuthmann, Richter, & Kliegl, 2005; Reichle, Liversedge, Pollatsek, & Rayner, 2009; Reichle, Pollatsek, & Rayner, 2006; Reilly & Radach, 2006; Snell, Declerck, & Grainger, 2018a; Snell, Meeter, & Grainger, 2017b; Snell, Vitu, & Grainger, 2017a). It should be noted that classic implementations of the flanker paradigm did not provide evidence for parallel processing. Following a serial processing rationale, participants could process the flankers after the processing of the target but before the final decision (given that flankers and target stayed onscreen until a response was given), thus leaving the flankers ample time to influence the response. However, more recent implementations trade on the use of very brief (150–170 ms) stimulus presentation times. Given that it takes around 150–250 ms to recognize a single word, and that the flankers will logically have disappeared once processing of the target is completed, here it can be argued that the flankers must be processed *during*, rather than *after,* target processing, if they are to have any influence on the response.

This is precisely what Dare and Shillcock (2013) demonstrated: using a 150-ms stimulus presentation time, lexical decisions about central target stimuli were influenced by the orthographic relatedness of adjacent letters, such that *rock* was recognized faster in “*ro rock ck*” than in “*st rock ep*” (see Grainger, Mathôt, & Vitu, 2014; Snell, Bertrand, Meeter & Grainger, 2018c; Snell, Vitu, et al., 2017a, for similar findings). These so-called parafoveal-on-foveal influences have also been established at higher cognitive levels. Snell, Meeter, et al. (2017b) found that the syntactic categorization of target words was influenced by the syntactic congruency of flanking words, and similarly, that the semantic categorization of target words was influenced by the semantic relatedness of flanking words (Snell, Declerck, et al., 2018a). Further, Declerck, Snell, and Grainger (2017) found that lexical decisions about target words were made faster when the targets were flanked by unrelated words of the same language, compared with unrelated words of a different language.

Point of controversy is the fact that similar higher-order parafoveal-on-foveal influences have been elusive in more natural reading settings, such as sentence reading. While some studies have reported semantic parafoveal-on-foveal effects in Chinese (Yan, Richter, Shu, & Kliegl, 2009; Yan & Sommer, 2015), similar effects were not found in Roman-alphabetic languages (Angele, Tran, & Rayner, 2013; Snell, Meeter, et al., 2017b, Snell, Declerck, et al., 2018a), save for one study that did not control for the orthographic overlap between targets and adjacent words (Inhoff, Radach, Starr, & Greenberg, 2000).^1^

This discrepancy between the flanker paradigm and sentence reading is likely caused by the different natures of the measures of interest in each respective setting. Sentence-reading research is concerned with word viewing times and eye movements (as observed with eye-tracking apparatus), which are thought to provide more direct measures of word recognition speed (e.g., Rayner, 1998). In contrast, a syntactic or semantic categorization decision, as measured in the flanker paradigm, likely takes place postlexically (Snell, Declerck, et al., 2018a; Snell, Meeter, et al., 2017b). The fact that higher-order cross-word influences seem to occur exclusively at this stage points to the possibility that words are truly recognized in parallel (i.e., without influencing one another at earlier levels of semantic or syntactic processing). Independently recognized words would then move on to jointly influence responses in the flanker paradigm, hence explaining the effects in RTs but absence of effects in word viewing times.

This reconciliation of contrasting behavioral outcomes in, respectively, the flanker paradigm and sentence reading, posits that these settings may be describing two sides of the same theoretical coin. Whereas sentence-reading research provides a good description of natural reading behavior, the flanker paradigm is suited to assess the reading system at a more fundamental level, hence revealing what the system is in principle capable of (e.g., parallel word processing).

Nonetheless, it would be sensible to assume that behavioral outcomes may not only differ because the respective measures of interest (word viewing times vs. lexical decision times) represent different things, but also because the reader approaches each respective task with different objectives. Whereas the primary goal when reading normal text is to achieve context comprehension, a lexical decision task is likely to induce a strategy that relies more on bottom-up processing of the visual input and less on top-down expectations (e.g., word identities being constrained by preceding context). Furthermore, sentence reading demands an efficient coupling of lexical processing and oculomotor control (e.g., Rayner, 1998), whereas a lexical decision task does not. It remains to be seen to which extent such factors dictate how other components of the reading system operate, and indeed, whether the interplay of cognitive processes such as visual processing, working memory, and attention is fundamentally different across tasks. In short, methodological differences between the flanker paradigm and sentence reading necessitate clarification of the extent to which cognitive processes underlying reading operate similarly in these respective settings.

## Investigating attention in the flanker paradigm

Considering the above, it is noteworthy that parafoveal-on-foveal effects of orthographic relatedness are observable in the flanker paradigm and sentence reading alike. In sentence reading, word viewing times are shorter when followed by an orthographically related word, compared with an unrelated word (Angele et al., 2013; Dare & Shillcock, 2013; Snell, Vitu, et al., 2017a).

One recent implementation of the flanker paradigm points to a widespread distribution of visuospatial attention as the key factor driving orthographic parafoveal-on-foveal effects. In their study, Snell, Mathôt, Mirault, and Grainger (2018b) manipulated the brightness of flanker locations, making use of the principle that the pupil size responds to the brightness of covertly attended (i.e., without looking) locations in the visual field (e.g., Mathôt & van der Stigchel, 2015). They found that target processing was influenced by flankers presented left and right of the target, but not by flankers presented above and below the target. Perfectly in line with this pattern, the pupil size was contingent with the brightness of horizontally but not vertically aligned flankers. This suggested that attentional resources were spent on the flankers involved in the parafoveal-on-foveal effect, hence evidencing a key role of attention. The widespread distribution of attention is in line with how parallel processing models (e.g., Engbert et al., 2005; Snell, Meeter, et al., 2017) conceptualize attention in sentence reading, and further illustrates the flanker paradigm’s merit in addressing questions about reading.

To test whether the flanker paradigm indeed provides an effective analogy to natural reading with respect to the attentional distribution, here we reason the other way around: Given that attention during sentence reading is biased toward the right in scripts that read from left to right (e.g., McConkie & Rayner, 1976; Rayner, 1998), there should similarly be a stronger influence from rightward than leftward flankers. This is not an obvious prediction: In classic variants of the flanker paradigm using single letter targets and flankers, a leftward bias has been observed (Harms & Bundesen, 1983; Hommel, 1995, 2003). However, Hommel (2003) showed that when flanker letters are mirrored, the bias is mirrored as well. This indicates that the attentional distribution is heavily dependent on the nature of the stimuli. In this light, a rightward attentional bias in a flanker task using linguistic stimuli could suggest that the reading system is triggered to engage in “real reading.” Preliminary evidence for such a rightward bias was provided by Snell, Bertrand, and Grainger (2018c), who found that RTs in a lexical decision task decreased when the bigram frequency of rightward flankers increased, while the bigram frequency of leftward flankers had no influence.

Experiment 1 provides a more complete test of the hypothesis that attention should be biased toward the right in the flanker paradigm. We measure lexical decision times for foveal targets as a function of seven flanker conditions that allow both for a replication of previously reported parafoveal-on-foveal effects, as well as an assessment of location-specific (left/right) flanker influences. All data reported in this this are openly accessible on https://osf.io/dbjvh/.

## Experiment 1

### Method

Thirty-five students (27 females) from Aix-Marseille University, ranging in age between 18 and 24 years, gave informed consent to participating in this study for 5 euros. All participants reported to be nondyslexic, native to the French language, and having normal or corrected-to-normal vision.

From the French Lexicon Project database (Ferrand et al., 2010) we retrieved 60 four-letter target words that contained no diacritics. For each target we retrieved an orthographically unrelated word (to be used as unrelated flanker) with the same length and frequency. In a similar fashion, we retrieved 60 nonword targets and unrelated nonword flankers from the Pseudo-Word Lexicon (Ferrand et al., 2010).

Each target was presented across seven conditions, visualized in Table 1. Each participant saw all 60 targets in all conditions, meaning that there were 420 word target trials per participant. For nonword targets (which were included solely to induce the lexical decision task, and data for which are thus not reported), we implemented the same flanker conditions, thus bringing the total amount of trials per participant to 840. These were presented in random order.

**Table 1:** Experiment 1 conditions

Condition	Left flanker	Target	Right flanker
No flankers		rock	
Repetition flankers	rock	rock	rock
Unrelated flankers	step	rock	step
Left repetition flanker	rock	rock	
Right repetition flanker		rock	rock
Mixed flankers, left rep.	rock	rock	step
Mixed flankers, right rep.	step	rock	rock

Participants were seated in a comfortable chair in a dimly lit room. The experiment, implemented with OpenSesame (Mathôt, Schreij, & Theeuwes, 2012), was shown on a 17-inch, 1,024 × 768 pixel, 150-Hz display, at such a distance from the participant that each character space in the stimulus subtended 0.3° of visual angle. Participants were given task instructions both by the experimenter as well as visually onscreen.

**Fig. 1:**
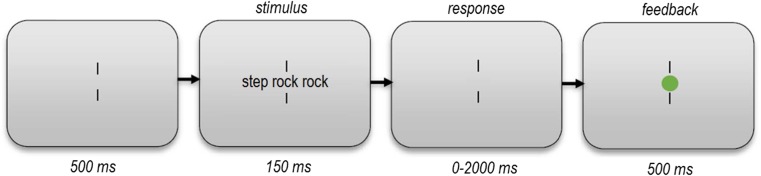
Trial procedure. The size of stimuli relative to the screen is exaggerated in this example

The trial procedure is shown in Fig. 1. Each trial started with a 500-ms fixation display containing centrally positioned vertical fixation bars, separated from one another by 0.60° of visual angle. A target stimulus (with/without flankers being presented on the left/right, depending on the condition as specified in Table 1) was shown for 150 ms in between the fixation bars, after which participants had a maximum of 2,000 ms to respond. Flankers were separated from the target by a single character space. Responses were given by means of a joystick trigger-button press with the right or left index finger, to indicate *word* or *nonword,* respectively*.* Providing response feedback, a green or red fixation dot was then shown at the center of the screen for 500 ms, for correct and incorrect responses, respectively (nonresponses were also counted as incorrect). The display then returned to the beginning state, 500 ms after which a new trial would commence. The experiment lasted approximately 35 minutes. The participants were offered a break after every 210 trials.

### Results

The analysis of RTs excluded incorrectly answered trials (6.69%). Additionally, both the analysis of RTs and the analysis of error rates excluded trials with an RT beyond 2.5 standard deviations from the grand mean (2.71%).

Data were analyzed using linear mixed-effect models (LMMs), with items and participants as crossed random effects (Baayen, Davidson, & Bates, 2008). The maximal random effects structure that converged was one including the by-subject random slope alongside by-participant and by-item random intercepts. However, a likelihood-ratio test pointed out that this model differed nonsignificantly from a model including only random intercepts. Following the recommendation of Baayen et al. (2008) that models not be overparameterized, we therefore did not include random slopes in the model. We report *b* values, standard errors (*SE*s) and *t* values (RTs) or *z* values (errors), with *t* and *z* values beyond |1.96| deemed significant.^2^

**Fig. 2:**
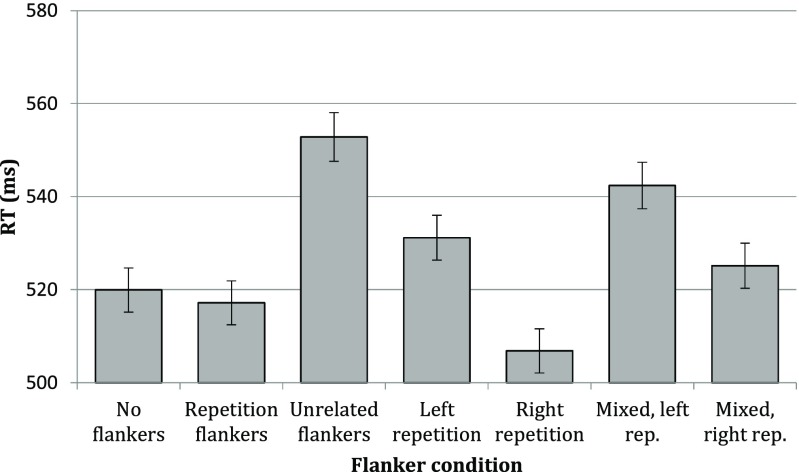
Mean RTs per condition in Experiment 1. Error bars depict 95% confidence intervals

Mean RTs per condition are presented in Fig. 2. We replicated previous observations of parafoveal-foveal integration, as the unrelated flanker condition yielded significantly longer RTs than did all other conditions (see Table 2). Our hypothesis that rightward flankers should have a stronger influence was confirmed: Participants benefitted more from a repetition flanker on the right (the fifth and seventh column in Fig. 2) than on the left (the fourth and sixth column). Between the mixed flanker conditions, a rightward repetition flanker yielded shorter RTs than a leftward repetition flanker, with *b* = −17.72, *SE* = 3.13, *t* = −7.40.^3^ When using a unilateral repetition flanker (the fourth and fifth column), RTs were again shorter for flankers presented on the right than on the left, with *b* = −24.66, *SE* = 3.05, *t* = −8.08.

**Table 2:** Analyses of RTs and errors in Experiment 1, with unrelated flankers as reference

Condition	*RTs*			*Errors*		
	*b*	*SE*	*t*	*b*	*SE*	*z*
(Intercept)	554.27	10.29	**39.39**	2.89	0.17	**17.28**
No flankers	−32.72	3.10	**−10.57**	0.12	0.12	0.96
Repetition flankers	−35.69	3.08	**−11.60**	−0.50	0.13	**−3.72**
Left repetition	−22.11	3.10	**−7.13**	−0.03	0.12	−0.21
Right repetition	−46.68	3.07	**−15.19**	−0.54	0.14	**−3.98**
Mixed flankers, left rep.	−9.38	3.12	**−3.01**	0.15	0.11	1.23
Mixed flankers, right rep.	−27.24	3.09	**−8.81**	−0.27	0.13	**−2.14**

Significant values are shown in bold

Strikingly, compared with the condition without flankers, a leftward repetition flanker yielded longer RTs (*b* = 10.70, *SE* = 3.08, *t* = 3.48), while a rightward repetition flanker yielded shorter RTs (*b* = −13.96, *SE* = 3.05, *t* = −4.58). This indicates that the recognition process may be truly facilitated by information on the right, while information on the left generally interferes. This would then also explain why the repetition flanker condition, which is basically a combination of the two unilateral flanker conditions, sits neatly in between these conditions. Indeed, no difference was observed between the repetition flanker condition and the condition without flankers (*b* = 2.97, *SE* = 3.07, *t* = 0.97), suggesting that the flanker on the right compensated for the flanker on the left.

It should be noted that while rightward parafoveal-foveal integration is clearly enhanced, there is nonetheless leftward integration, as the leftward repetition in the mixed flanker condition led to shorter RTs than the unrelated condition did: *b* = −9.38, *SE* = 3.12, *t* = 3.01.

Further illustration of the stronger rightward influence is that compared with the unrelated flanker condition, errors were significantly reduced if a repetition flanker was presented on the right, regardless of what was presented on the left (see Table 2).

### Discussion

Experiment 1 provides clear evidence for a rightward attentional bias in a flanker paradigm using linguistic stimuli. While readers benefitted from a repetition flanker on the left compared with an unrelated flanker (evidenced by contrasting the mixed flanker condition with the unrelated flanker condition), this difference was clearly enhanced for rightward flankers.

Compared with a condition without flankers, a unilateral repetition flanker further led to better performance when being presented on the right, while its presentation on the left led to worse performance. This particular finding is quite striking, as it illustrates that parafoveal-related information can truly facilitate foveal word processing—on the premise that the related information is located in the favored hemifield. Below, we report a second experiment with which we intended to replicate this finding. Experiment 2 tested the two unilateral repetition flanker conditions and the no-flanker condition alongside two conditions with unilateral unrelated flankers.

## Experiment 2

### Method

Of the 35 participants that were recruited for Experiment 2 (32 females, age range 18–29 years), 10 had also participated in Experiment 1.^4^ Aside from testing a different set of conditions (presented in Table 3), the entire methodology for Experiment 2 was unchanged from that of Experiment 1. Including nonword targets, Experiment 2 comprised 600 trials, spanning approximately 25 min of testing time per participant.

**Table 3:** Experiment 2 conditions

Condition	Left flanker	Target	Right flanker
No flankers		rock	
Left repetition flanker	rock	rock	
Right repetition flanker		rock	rock
Left unrelated flanker	step	rock	
Right unrelated flanker		rock	step

### Results and discussion

All criteria for data selection and analysis were equal to those of Experiment 1. There were 7.41% of trials excluded from the analysis of RTs due to either an incorrect response or an RT beyond 2.5 standard deviations from the grand mean. The latter criterion led to the exclusion of 2.44% of trials from the error rate analysis.

**Fig. 3:**
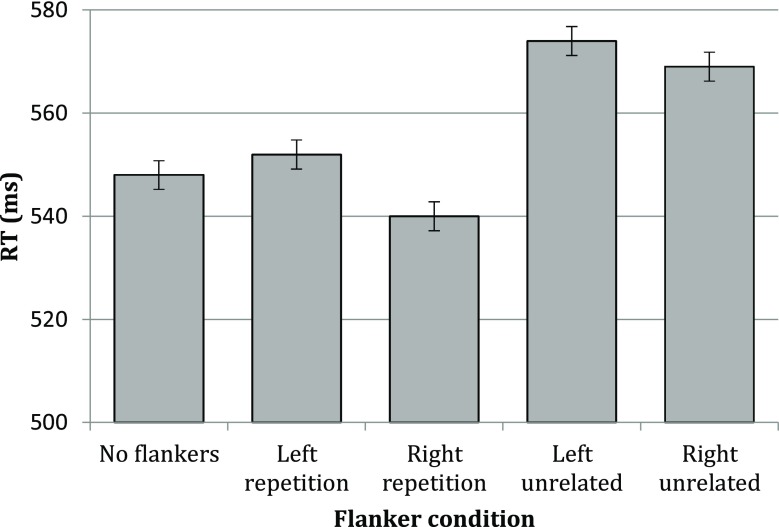
Mean RTs per condition in Experiment 2. Error bars depict 95% confidence intervals

As can be seen in Fig. 3, we replicated effects observed in Experiment 1, with a rightward repetition flanker leading to shorter RTs than the no-flanker condition. However, whereas the leftward repetition flanker yielded longer RTs than the no-flanker condition did in Experiment 1, we observed no significant difference between these conditions this time (see Table 4). That the presence of a leftward flanker induced a smaller cost in Experiment 2 compared with Experiment 1 is possibly because a greater portion of trials presented flankers exclusively on the left in Experiment 2 compared with Experiment 1 (i.e., 40% of trials in Experiment 2 vs. 14.29% of trials in Experiment 1), which may have led participants to attend the left visual hemifield to a greater degree. Nonetheless, Experiment 2 clearly replicated the observation of a processing asymmetry indicative of a rightward attentional bias.

**Table 4:** Analyses of RTs and errors for Experiment 2, with the no-flanker condition as reference

Condition	*RTs*			*Errors*		
	*b*	*SE*	*t*	*b*	*SE*	*z*
(Intercept)	553.34	10.80	**37.36**	3.13	0.17	**18.35**
Left repetition flanker	3.23	2.96	1.09	0.06	0.13	0.45
Right repetition flanker	−9.89	2.93	**−3.38**	−0.56	0.15	**−3.68**
Left unrelated flanker	24.51	2.96	**8.29**	0.05	0.13	0.37
Right unrelated flanker	20.23	2.96	**6.85**	0.02	0.13	0.18

Significant values are shown in bold

Noteworthy is that the attentional bias not only leads to overall decreased RTs with rightward flankers compared with leftward flankers but also enhances the parafoveal-on-foveal effect of orthographic relatedness for information in the attended region. To support this notion, we analyzed the four unilateral flanker conditions in a separate model that included flanker relatedness (*repetition, unrelated*) and flanker laterality (*left, right*) as factors. In addition to the main effects of relatedness (*b* = −21.20, *SE* = 2.97, *t* = −7.15) and laterality (*b* = −13.18, *SE* = 2.94, *t* = −4.49), we indeed also established an interaction between these factors: *b* = 8.97, *SE* = 4.17, *t* = 2.15, such that the effect of orthographic relatedness was increased for rightward flankers. This further supports the claim of Snell Mathôt, et al. (2018b) that the spatial integration of orthographic information is driven by attention.

The error rate was again only influenced by the relatedness of rightward flankers (see Table 4). Indeed, the interaction between relatedness and laterality that was established for RTs was also established for error rates: *b* = 0.59, *SE* = 0.20, *z* = 2.98, such that the effect of relatedness on the error rate was greater for rightward flankers than for leftward flankers.

## General discussion

Across two experiments, we observed a strong rightward processing bias in a flanker paradigm using linguistic stimuli. Flanker relatedness being equal, leftward flankers consistently led to longer RTs than did rightward flankers. As Snell, Mathôt et al. (2018b) have shown that spatial orthographic integration effects are driven by attention, we therefore conclude that attention in a flanker paradigm with word stimuli is biased toward the right. This processing asymmetry contradicts the leftward bias observed in classic implementations of the Eriksen flanker task (e.g., Harms & Bundesen, 1983; Hommel, 1995, 2003) and instead parallels the rightward bias observed in natural (sentence) reading (McConkie & Rayner, 1976; Rayner, 1998).

Evidenced by both Experiments 1 and 2, word processing is facilitated by the presence of the same word when the latter is located within the attended region of the visual field. This benefit is negated (Experiment 2) or even turns into a cost (Experiment 1) when the repetition is presented outside the locus of attention. We reason that a leftward shift of attention, triggered by the leftward stimulus, shifted some processing resources away from the target, hence leading to longer RTs.

This implies that parafoveal words can impact on foveal word processing in two ways: first, information is integrated across words, such that related parafoveal information leads to faster foveal word recognition compared with unrelated parafoveal information (a pattern observed for both leftward and rightward flankers). Building on the study of Snell, Mathôt et al. (2018b), which established that attention is a key factor driving the spatial integration of orthographic information, here we observed that an attentional bias enhances integration effects at the attended region (see Experiment 2, Results and Discussion).

The second way in which parafoveal words can impact on foveal word processing, is purely attentional: When presented outside the attended region of the visual field, exogenous attentional capture by the flanker shifts some processing resources away from the target. Following this logic, if a flanker is presented within the attended region, then the spatial integration of information should be the only factor determining the flanker’s impact on target processing, hence, explaining the facilitatory influence of rightward related flankers. In contrast, a leftward flanker induces a shift of attention, and therefore less efficient target processing—although this may be compensated to some extent by the spatial integration process.

The above scenario generates predictions that may be addressed in future research. If attention is biased toward the left *prior* to the onset of the target and flankers—for instance by a directional/spatial cue (e.g., Posner, 1980)—then the pattern of effects observed here should be mirrored. We thus predict facilitation of repetition flankers presented at the cued location, and interference from (both repetition and unrelated) flankers presented opposite to the cued location. The effect of parafoveal-foveal relatedness should further be enhanced at the cued side.

It must be acknowledged that the rightward bias of attention may not be the only factor driving the observed processing asymmetry. Prior research has shown that the recognition of isolated words is better in the right than in the left parafovea (e.g., Ducrot & Grainger, 2007), and one factor that likely contributes here is the fact that language is typically processed in the left hemisphere. As argued by Brysbaert (2004), asymmetries in visual language processing may stem from the fact that information in the right visual hemifield is processed by a shorter neural pathway (i.e., from the left visual cortex to the left-hemispheric language centers) than information in the left visual hemifield, the neural pathway for which additionally comprises interhemispheric transfer through the corpus callosum. However, Ducrot and Grainger (2007) found that spatial cues affected the rightward processing bias, thus suggesting that attention nonetheless plays a role as well. To pinpoint the respective contributions of the attentional bias and hemispheric asymmetry to the observed processing asymmetry, future research may compare conditions with unilateral unrelated flankers to conditions with unilateral nonlinguistic flankers (e.g. mask flankers: ####).

The present results have three implications for reading research. First, it is apparent that readers’ distribution of attention can be quite typical of natural reading, even outside a natural reading setting. This advances the conception that the flanker paradigm is suited to test aspects of the reading process—at least when pertaining to attention—in a simple and controlled setting that does not necessitate the use of eye-tracking apparatus.

Secondly, the present results generate certain predictions for the realm of sentence reading. Specifically, these results predict that orthographic parafoveal-on-foveal effects from word *n* − 1 on *n* should be observable in sentence reading, even if influences from word *n* + 1 should nonetheless be stronger.

Thirdly, particular care is warranted when employing the flanker paradigm to test within-word letter processing. For instance, Snell, Bertrand, et al. (2018) turned the principle that information is integrated across words into an asset for investigating how letter position is encoded (using various configurations of the target’s letters as flankers to see how these affect the recognition process; see also Grainger et al., 2014). In such an experiment, flanker letters may be expected to yield different outcomes depending on laterality.

At the same time, taking the processing asymmetry into account can lead one to gain additional information. As an illustration, consider the studies of Dare and Shillcock (2013) and Grainger et al. (2014), both of which found that word processing was facilitated not only by related bigram flankers presented on the correct side of the target (e.g., “*ro rock ck*”) but equally as much by switched bigram flankers (“*ck rock ro*”). Knowing that the parafoveal-on-foveal effect will largely have been effectuated by different letters in the former condition (“*ck*”) compared with the latter condition (“*ro*”), one may conclude that, within words, beginning and ending letters bear equal importance. Note that one could not have concluded this without knowing about the processing asymmetry: If flanker processing were symmetrical, then the summed facilitation of “*ro*” and “*ck*” would always be equal to the summed facilitation of “*ck*” and “*ro*,” even if ”*ro*” bore more weight than “*ck*” (forasmuch as 5 + 1 equals 1 + 5), implying that one could not retrieve each bigram’s respective importance in this case.^5^

In sum, having revealed a rightward attentional bias in the flanker paradigm, the present study attests to the conception that the paradigm provides an effective analogy to natural reading. Future research employing the flanker paradigm should take this asymmetry into account.

## Footnotes

1 Note that this is not to say that word viewing times are not affected by postlexical processes (e.g., context comprehension) during normal reading. Rather, we specifically note that eye-movement measures relating to word *n* (i.e., fixation durations on *n*, skipping rate of *n*, the number of refixations on *n,* and regressions to *n*) are largely unaffected by higher-order (semantic, syntactic) aspects of word *n* + 1.

2 While the statistical analyses reported were performed on normal RTs, some have argued that log-transformed RTs would better fit the distributional assumptions of the model (e.g., Kliegl, Wei, Dambacher, Yan, & Zhou, 2010). Following advice by a reviewer of this work, we have performed the same analyses on log-transformed RTs and established virtually equal significance values across the two data sets.

3 Note that these values were obtained by choosing the leftward repetition flanker condition as the reference in the original model. Values reported elsewhere in the text were similarly obtained by re-referencing the model.

4 A model including subjects’ prior experience (*did* or *did not participate in Experiment 1*) as a factor pointed out that subjects’ prior experience did not modulate the pattern of reported effects. There was no main effect of prior experience (*b* = −28.88, *SE* = 23.02, *t* = −1.25), no interaction with flanker relatedness (*b* = −1.70, *SE* = 6.53, *t* = −0.26), or flanker laterality (*b* = −0.36, *SE* = 6.45, *t* = −0.06), and no three-way interaction of these three factors (*b* = 4.20, *SE* = 9.19, *t* = 0.46).

5 Note that the studies of Grainger et al. (2014) and Dare and Shillcock (2013) are only used here for illustrational purposes; after all, it is possible that participants did not have a rightward attentional bias in these studies, because they saw two-letter flankers rather than whole-word flankers (and were thus possibly not as much engaged in “real reading”).
